# A clinico‐pathological study of geriatric anemias

**DOI:** 10.1002/agm2.12150

**Published:** 2021-05-05

**Authors:** Amit H. Agravat, Krupal Pujara, Ravi Kiritkumar Kothari, Gauravi A. Dhruva

**Affiliations:** ^1^ Pandit Deendayal Upadhyay Medical College Rajkot India

**Keywords:** anemia of chronic disease, elderly, normocytic normochromic anemia

## Abstract

**Introduction:**

Anemia in the older age (e.g., >60 years) is a major health problem in India and many parts of the world since it signifies an underlying disease and is associated with poor clinical outcome like increased morbidity and affects health‐related quality of life. Since symptoms like fatigue or shortness of breath related to anemia could also be attributed to the aging process, anemia is often easily overlooked in the elderly.

**Aims and objectives:**

Clinico‐hematological patterns and morphological types of anemia in older age (e.g. >60 years) are manifold, hence this study was undertaken to determine them and to know more about associated disorders.

**Materials and methodology:**

The present study was conducted on a sample size of 1257 patients who were 60 years and above and clinically diagnosed as anemic. Routine haematological investigations including peripheral blood smear examination and complete hemogram were done. Special investigations like bone‐marrow examination and iron studies were done whenever required.

**Results:**

Males (aged >60 years) were more affected than females (aged >60 years) and patients in the age group of 60–69 years were affected the most. The most common presenting symptom was generalized weakness. The most common morphological type was normocytic normochromic anemia, and chronic diseases were the commonest etiological factors.

**Conclusion:**

In spite of modern diagnostic advances, geriatric anemias still remain under‐reported and inadequately investigated, necessitating evaluation of even mild anemias. Prompt diagnosis and definite categorization helps in appropriate management of anemias.

## Graphical abstract

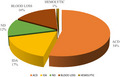

## 1 INTRODUCTION

Anemia is a global health problem in the older adult population because of the high prevalence and associated significant morbidity and mortality.^1^, ^2^ It is easy to overlook anemia in the elderly since symptoms like fatigue, weakness, or shortness of breath could also be attributed to the aging process itself and will never be accepted as an inevitable consequence of aging. The reported prevalence of anemia in the elderly is 2.9%–51% and correlates with advanced age and multiple related conditions, including iron deficiency, inflammatory conditions, malignancy, and low serum erythropoietin.^2^ Thus, anemia in elderly patients is an emerging global health problem for the 21st century that negatively impacts the quality of life.^2^ Aging by itself is unlikely to cause anemia. Hemoglobin levels within healthy older individuals don't change significantly from 60 to 98 years of age. Changes that occur commonly during aging increase the risk of anemia, thus explaining the association of anemia with older age. These include reduced ability to absorb essential nutrients, decreased hematopoietic reserve, and reduced sensitivity to erythropoietin.^3^

## 2 OBJECTIVES

- To study the clinico‐hematological patterns of anemia in elderly patients 60 years and above.
- To detect the morphological types of anemia prevalent amongst them.
- To know common etiology for anemia.
- To know various associated disorders.

## 3 MATERIALS AND METHOD

### 3.1 Inclusion criteria

1. Indoor Patients at PDU Hospital.
2. Patients with age > 60 years.
3. Patients having Hb <12 gm/dL.

### 3.2 Exclusion criteria

1. OPD Patients at PDU Hospital.
2. Patients with age < 60 years.
3. Patients having Hb >12 gm/dL.

The present study is a descriptional cross‐sectional study that was conducted over 5 years, that is, August 1, 2015–October 31, 2020. All indoor patients who were 60 years and older and clinically diagnosed as anemic were included. Routine haematological investigations. Peripheral blood smear examination using Field stain and Leishman stain. Special investigations like iron studies, reticulocyte count, Perl’s Stain, and bone‐marrow examination were done whenever required.

## 4 OBSERVATIONS AND ANALYSIS

Table 1 indicates that the maximum number of subjects (1013) were in the age group of 60–70 years, 179 subjects were in the age group of 71–80 years, and 65 were subjects in the age group of 80 years and above.

**TABLE 1:** Age wise distribution of cases

Age group (years)	Total number (n = 1257)	Percentage (100%)
60–65	784	62.4%
66–70	229	18.2%
71–75	97	7.7%
76–80	82	6.5%
81‐85	49	3.9%
85‐90	16	1.3%
Total	1257	100%

**FIGURE 1:**
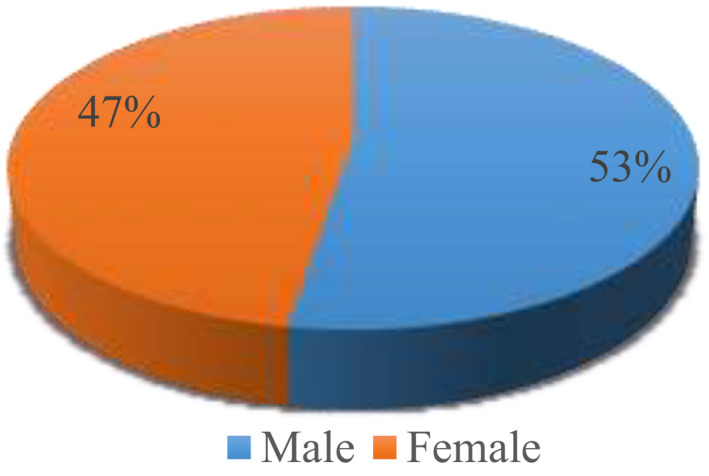
Pie diagram representing gender wise distribution of cases

**FIGURE 2:**
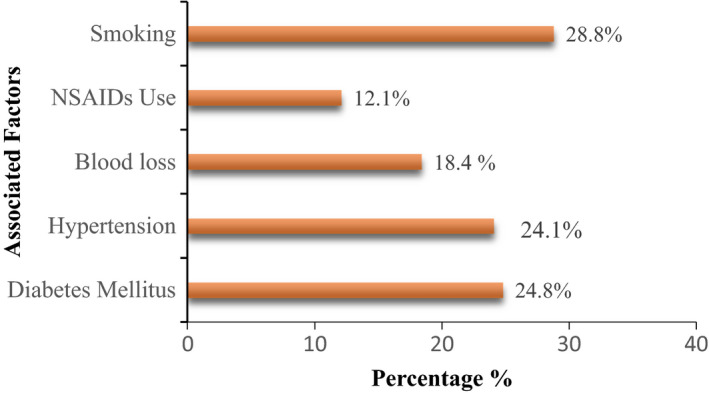
Correlation with associated factors

Figure 1 shows that 52.6% subjects were male and 47.4% were female in the present study (Figure 2).

In the present study, 28.8% of the patients were smokers, 24.1% had hypertension, 24.8% had diabetes mellitus, 18.4% had blood loss, and 12.1% were using NSAIDS (Table 2).

**TABLE 2:** Relation with symptoms and sign

Associated factor	Total number n = 1257	Percentage (100%)
Respiratory	264	21%
Gastrointestinal	182	14.5%
Carcinomas	176	14%
Nutritional disorders	164	13%
Liver	88	7%
Renal	94	7.5%
Non specific	289	23%
Total	1257	100%

In this study, non‐specific symptoms were most commonly associated with anemia, followed by symptoms and signs of respiratory illness, gastrointestinal diseases, carcinoma, nutritional disorders, liver and renal diseases (Table 3).

**TABLE 3:** Peripheral blood smear patterns

Peripheral blood smear findings	Total number n = 1257	Percentage 100%
Normocytic Normochroimc anemia	566	45.0%
Hypochroic microcytic anemia	369	29.4%
Dimorphic anemia	210	16.7%
Normocytic hypochromimc anemia	68	5.4%
Macrocytic anemia	44	3.5%
Total	1257	100%

**FIGURE 3:**
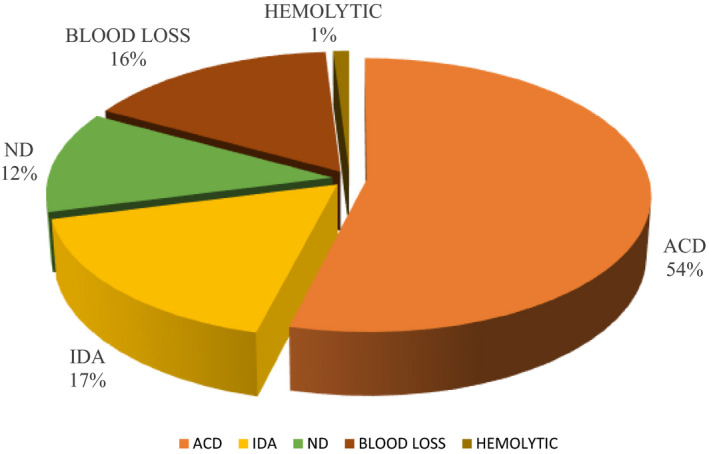
Pie diagram representing causes of Anemia. ACD, anemia of chronic disease; IDA, iron deficiency anemia; ND, nutritional deficiency anemia.

In the present study, we observed that the most common morphological type of anemia was normocytic normochromic (45%) followed by hypochromic microcytic (29.4%), dimorphic (16.7%), normocytic hypochromic anemia (5.4%), and macrocytic (3.5%), which was the least common (Figure 3).

We observed in the present study that anemia due to chronic disease (54%) was the most common type followed by iron deficiency anemia (17%), anemia due to other nutritional deficiencies (12%), anemia due to blood loss (16%), and the least common was anemia due to hemolysis (1%).

**FIGURE 4:**
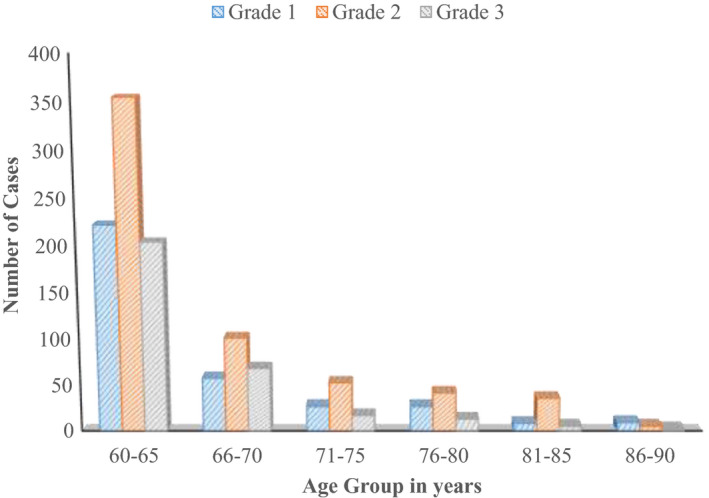
Age group wise grading of anemias

Figure 4 shows that out of 1257 patients 594 had Grade 2 anemia (moderate anemia).

## 5 DISCUSSION

In the present study, more males were found to be anemic as compared to females. A similar gender‐wise distribution was noted within the studies by Khatib et al^4^ and Ghandhi et al.^5^Our result differs from Abhishek Pathania et al and studies by Amarneel et al,^6^ Joosten et al,^7^ and Tay and Youong^8^ in which females were more anemic (Tables 4 and 5).

**TABLE 4:** Comparative study of gender wise distribution of geriatric anemias

Gender	Present study (Rajkot) [2020] n = 1257	Abhishek Pathania et al (AIIMS New Delhi) [2019] n = 229	Samarneel et al (Bhavnagar Gujarat) [2015] n = 42	Khatib et al (Karad, Maharashtra [2016] n = 256	Ramya et al (Puduchhery) [2016] n = 675	Joosten et al (Belgium) [1992] n = 178	Mathew Tay et al (Singapore) [2011] n = 424
Male	**52.6%**	36.7%	28.6%	**53.9%**	**51%**	38.8%	48.6%
Female	47.4%	**63.3%**	**71.4%**	46%	49%	**61.2%**	**51.4%**

Bold indicates majority of cases.

**TABLE 5:** Comparative study of maximally affected age group

Age group (years)	Present study (Rajkot, Gujarat) 2020 n = 1257	Amarneel et al (Bhavnagar, Gujarat) 2015 n = 42	Nisha et al (Kozhikode, Kerala) [2017] n = 826	Kiran et al (Dharwad, Karnataka) [2017] n = 100	Geisel et al (Germany) [2017] n = 388
50‐59	–	–	18.3%	–	–
**60‐70**	**80.6%**	**61.25%**	**44%**	**70%**	13.9%
71‐80	14.2%	27.5%	26.8%	23%	40.2%
81‐90	5.2%	11.25%	10.8%	7%	**46%**

Bold indicates majority of cases.

In the present study, patients in the age group of 60–70 years were maximally affected, which is in concurrence with the studies by Amarneel et al,^6^

Nisha et al,^9^ and Kiran et al,^10^ whereas in a study done by Geisel et al,^11^ patients in the age group of 81–90 years were maximally affected (Table 6).

**TABLE 6:** Comparative study of contributory causes resulting in anemia

Cause of Anemia	Present study (Rajkot, Gujarat) [2020] n = 1257	Nisha et al (Kozhikode, Kerala) [2017] n = 500	Guyatt et al (Ontario, Canada) [1990] n = 259	Joosten et al (Belgium) [1992] n = 178	Tay et al (Singapore) [2011] n = 424
Iron deficiency anemia	16.8%	12.2%	36.3%	15%	13%
Anemia of chronic disease	**54**%	**48.9%**	**43.6%**	**41.5%**	**29.3%**
Nutritional anemia	12%	6.9%	8.10%	5.5%	13%
Blood loss	15.7%	8.5%	–	7.0%	–
Hematologicalmalignancy	0.8%	18.5%	2.70%	11%	0.7%
Others	0.7%	5%	9.3%	20%	44%

Bold indicates majority of cases.

In the present study, the most common underlying cause of anemia is anemia of chronic disease. This finding is in concurrence with the studies by Guyatt et al^12^ Nisha et al, and Joosten et al, in which chronic disease was maximally responsible for anemia followed by iron deficiency anemia. In hematological malignancy chronic myeloid, leukemia was present in 10 (0.8%) subjects in the present study, which correlates with Kiran et al^10^ in which 1% was noted. Chronic leukemia and lymphoproliferative disorder was noted in 0.4%, which differs from the study conducted by Nisha et al^9^ (9.7%) and Vijay Tailak et al^13^, 2.2 % have the anemia due to Chronic leukemia and lymphoproliferative disorder. In the present study myelodysplastic syndrome is present in 5 (0.4%) subjects, which is concurrent with the study done by Vijay Tailak et al^13^ showing 1.4% of subjects with myelodysplastic syndrome.

At least one‐third of anemic patients older than 65 years show a hyperinflammatory state typical for CKD or for AI (cancer, autoimmune disease, and chronic infection). Underlying pathophysiological mechanisms in AI are manifold, are overlapping, and show differences in extent between patients.

First, reduced EPO production that is too low to counteract anemia and a blunted response of erythroid progenitors to EPO represent essential underlying mechanisms.

A direct negative effect of different cytokines, like tumor necrosis factor α, interleukin‐1 (IL‐1), and transforming growth factor β, on proliferation and differentiation of erythroid progenitor cells has also been reported and is at least partly because of a downregulation of EPO receptor expression on erythroid progenitors. In addition, these cytokines promote myelopoiesis with the overall net effect of reduced erythropoiesis.^14^ Alterations in energy metabolism and body composition have also been reported to potentially regulate erythropoiesis in the elderly.

Second, an essential mechanism driving the development of AI is an increased uptake and retention of iron (in the form of senescent/damaged erythrocytes) within the reticuloendothelial system leading to an iron‐restricted erythropoiesis.

Hepcidin, a mainly liver‐derived antimicrobial acute phase protein, reduces both duodenal iron absorption and iron release from macrophages. These effects can be explained by the interaction of hepcidin and the transmembrane protein ferroportin, the only so far known iron exporter in mammalians. In macrophages, which have a general turnover of approximately 20–25 mg of iron per day as a result of being recycled from senescent red blood cells (RBCs), this produces iron restriction with an accompanying increase in ferritin levels and decrease in transferrin saturation (TSAT), resulting in a relative iron‐deficient erythropoiesis.

Increased hepcidin levels have been reported in cancer patients and patients suffering from autoimmune disease and CKD. Remarkably, elevated hepcidin levels have also been reported in older patients, with an age‐related increase. As hepcidin seems to be the central player in iron metabolism, several mechanisms are involved in tightly controlling hepcidin. Hepcidin expression is upregulated by inflammatory cytokines like IL‐6 and different bone morphogenic proteins (BMPs), mainly BMP6 and BMP2. Moreover, endoplasmic reticulum stress and reactive oxygen species (ROS), as well as reduced levels of estrogen and testosterone, seem to directly increase hepcidin expression. This helps in understanding why endocrine changes at menopause or andropause result not only in a constitutively increased presence of inflammatory mediators, but also in increased hepcidin levels.

Third, eryptosis, the phagocytosis of aging erythrocytes triggered by changes in their plasma membrane, is often discussed as a further hallmark in the development of AI. Recycling of aged and/or damaged RBCs occurs under physiological conditions mainly in the spleen. It is well known that in distinct situations including inflammation, RBC numbers and Hb levels drop much faster than can be explained by a pure reduction in RBC production and Hb synthesis. In fact, translocation of phosphatidylserine to the membrane surface is a first step in this process.^15^ It enables macrophages to engulf erythrocytes and ultimately eliminate them from circulation. Lupescu et al showed that ROS production leads to a much higher frequency of phosphatidylserine‐presenting erythrocytes in older than in younger patients. Other reports have shown that disorders that are quite common at advanced age, including dehydration, diabetes mellitus, or chronic heart disease, might also affect RBC stability (Table 7).

**TABLE 7:** Comparative study of grading of anemia

Grade of Anemia	Present study (Rajkot, Gujarat) [2020] n = 1257	Abhishek Pathania et al (AIIMS, New Delhi) [2019] n = 229	Nisha TR et al (Kozhikode, Kerala) [2017] n = 826	Suma JK et al (Mysore) [2013] n = 114	Ramya et al, (Puducherry) [2016] n = 675	Joosten et al (Belgium) [1992] n = 178
Mild (10–12 gm/dl)	28%	**47.60**	**68.8%**	**19.29%**	**80.9%**	29.2%
Moderate(7–10 gm/dl)	**47.26%**	47.16	26.3%	16.7%	16.7%	**57.9%**
Severe (<7 gm/dl)	24.74%	5.24	4.9%	2.4%	2.4%	12.9%

Bold indicates majority of cases.

In the present study, the highest number of subjects are with moderate degree (Grade II) of anemia. This finding is in concurrence with the studies by Suma et al and Joosten et al.

Our result differs from the studies conducted by Abhishek Pathania et al, Nisha TR et al^9^, and Ramya et al, in which the majority of the elderly had mild anemia (Grade I).

Anemia in the elderly is a significant universal problem that is associated with poor clinical outcome. Though it is a critical issue that needs to be addressed on a priority basis, especially in developed countries, it is most often overlooked or sidelined owing to the more pressing and demanding diseases in the elderly.

In the elderly patients in whom anemia has a high prevalence, neither the hemoglobin threshold nor the identity of the disease causing anemia is easily established. This is an important shortfall because even mild anemia can compromise a patient's well‐being and survival, regardless of the underlying cause.^16^

Anemia due to chronic diseases is the most common form of geriatric anemia as observed in the present study. This study is concurrent with the study by Mauro Tettamanti et al (Table 8).^17^

**TABLE 8:** Comparative study of associated co‐morbidities

Associated Comorbidities	Present study (Rajkot, Gujarat) [2020] n = 968	Suma et al Mysore [2013] n = 33	Kiran et al (Dharwad, Karnataka) [2017] n = 100	Tay et al (Singapore) [2011] n = 23	Geisel et al (Germany) [2017] n = 83
GI Disorder	18.8%	18.2%	–	8.69%	15.66
Liver	9.1%	6.0%	20%	21.73%	–
Renal	9.7%	12%	**50%**	–	**56.6%**
Respiratory	27.3%	**36.4%**	16.5%	**34. 8%**	–
Carcinoma	18.2%	15.2%	1%	34.8%	12.04
Arthritis	17.9%	12.1%	12.5	–	15.7

Bold indicates majority of cases.

In the present study respiratory disease is associated in most of the subjects (27.27%), which correlates with the studies conducted by Suma et al (36.4%) and Tay (34.8%); the next common condition associated was Gestro intestinal disorder. In hematological malignancy, chronic myeloid leukemia was present in 10 (0.8%) subjects in the present study and correlates with Kiran et al in which 1% was noted. Chronic leukemia and lymphoproliferative disorder was noted in 0.5%, which differs from the study conducted by Nisha et al^9^ having 9.7% and Vijay Tailak et al^13^ having 2.2% of chronic leukemia and lymphoproliferative disorder. Study myelodysplastic syndrome is present in 4 (0.3%) subjects concurrent with the study done by Vijay Tailak et al having 1.4% of subjects with myelodysplastic syndrome.

## 6 CONCLUSION

Despite the fashionable diagnostic advances, geriatric anemia still remains under‐reported and inadequately investigated, especially when mild, thereby necessitating evaluation of even mild anemias during this vulnerable population. Non‐specific symptoms like fatigue and weakness should not be ignored or attributed to the normal aging process as it can be an important signal to the presence of anemia. Improved definitions of anemia and more detailed investigations like bone marrow aspiration and biopsy also help to define the subtypes of anemia, thereby facilitating prompt and accurate diagnosis to ensure appropriate patient management.

## ACKNOWLEDGEMENTS

I Heartily thank Dr. Gauravi A Dhruva, Professor and head, Department of Pathology, Pandit Dindayal Upadhyay Medical College, Rajkot For her help, valuable advice and suggestions. Dr. Rutvi Teli my colleague for writing assistance.

## CONFLICTS OF INTEREST

Nothing to disclose.

## AUTHOR CONTRIBUTIONS

Dr. Amit Agravat, Dr. Krupal Pujara, and Dr. Ravi Kothari put forward the conceptualization and performed the data curation and formal analysis. Dr. Amit Agravat and Dr. Krupal Pujara wrote the original draft. Dr. Amit Agravat and Dr. Ravi Kothari wrote and edited the manuscript. Dr Gauravi Dhruva supervised the whole process.
